# Statement of Retraction: Microstructure and wear behaviour of AlCoCrFeNi-coated SS316L by atmospheric plasma spray process

**DOI:** 10.1080/14686996.2025.2552534

**Published:** 2025-09-01

**We, the journal office and Publisher of *Science and Technology of Advanced Materials (STAM)*, have retracted the following article:**

Noble, N., Radhika, N., & Natarajan, J. (2024). Microstructure and wear behaviour of AlCoCrFeNi-coated SS316L by atmospheric plasma spray process. Science and Technology of Advanced Materials, 25(1). https://doi.org/10.1080/14686996.2024.2341611

Following publication, the publisher was contacted by a third party with concerns about the integrity of the data, specifically:

  ●  Apparent similarities between Figure 2b in the above article and the image in Figure 3b of:

Radhika, N., Noble, N. & Adediran, A.A. Electrochemical and hot corrosion behaviour of annealed AlCoCrFeNi HEA coating over steel (2024). Scientific Reports 14(5652). https://doi.org/10.1038/s41598-024-55962-1

When contacted by the journal for an explanation, the authors were unable to address the concerns raised. As a result of these concerns, the STAM office no longer has confidence in the validity of the findings in the article, and therefore we are retracting the article from the journal. The corresponding author listed in this publication has been informed. The author does not agree with the retraction.

We have been informed in our decision-making by our editorial policies and the COPE guidelines.

The retracted article will remain online to maintain the scholarly record, but it will be digitally watermarked on each page as ‘Retracted’.

